# Supplementary material for: Assessing the impact of institutional mistrust on parental endorsement for COVID-19 vaccination among school communities in San Diego County, California
Source: PLoS One. 2024 May 28;19(5):e0295618. doi: 10.1371/journal.pone.0295618 (PMC11132501; doi:10.1371/journal.pone.0295618)
Supplement: Attachment — Submitted filename: Review of Manuscript - PONE-D-23-15837.docx [file pone.0295618.s001.docx]

**Review of Manuscript: PONE-D-23-15837**

This manuscript was to give information on the assessment of the impact of institutional mistrust on parental endorsement for COVID-19 vaccination among school communities. However, there are a few missing information that makes the outcome of the study inconclusive as reported.

In my opinion, there are a few things to consider to improve the manuscript

**Major:**

1. The authors determining the reasons or factors that may have prevented them from vaccinating their children or influence them in future vaccinations would have made a lot of difference. Even though this was stated in the study’s limitation, providing such information would have played a key role in making the final conclusion of the study very specific, confidently attributing the outcome of the study with institutional mistrust.

**Minor:**

2. The authors indicate obtaining a signed consent form from study participants. This should be clear, whether it was also part of the online survey or a separate written consent.
