# Supplementary material for: Assessing the impact of institutional mistrust on parental endorsement for COVID-19 vaccination among school communities in San Diego County, California
Source: PLoS One. 2024 May 28;19(5):e0295618. doi: 10.1371/journal.pone.0295618 (PMC11132501; doi:10.1371/journal.pone.0295618)
Supplement: Attachment — Submitted filename: Response to Reviewers.docx [file pone.0295618.s002.docx]

To the Editors:

Thank you for giving us the opportunity to resubmit our manuscript. We have edited our paper to address reviewer feedback; please see our comments below.

1. The use of secondary data in this study gives an interesting view. Mainly, primary studies have been used in such studies. The background of this study was however scanty and could not give a better explanation to the issues of misinformation.
   a. Thank you for your comment. The authors agree that misinformation is a big influence on COVID-19 vaccination uptake and institutional mistrust; however, sources of misinformation was not a primary outcome of the parent study. The study team made the decision to omit this type of item to reduce burden on study participants. Future research can build off from our study and assess this relationship.
2. The authors determining the reasons or factors that may have prevented them from vaccinating their children or influence them in future vaccinations would have made a lot of difference. Even though this was stated in the study’s limitation, providing such information would have played a key role in making the final conclusion of the study very specific, confidently attributing the outcome of the study with institutional mistrust.
   a. The authors agree that this information would have been very insightful for our analysis; however, as the reviewer points out in the above comment, this was a secondary analysis of survey data collected for a different study objective. Despite this, we believe that our findings are still relevant and important for public health action. Future research can build off from our study and explore individual motivations for COVID-19 vaccination for children.
3. The authors indicate obtaining a signed consent form from study participants. This should be clear, whether it was also part of the online survey or a separate written consent.
   a. Thank you for the feedback. We have edited a sentence in the *Ethics* section to clarify this. The sentence reads, “As part of the survey, participants were asked to review and sign a consent form before proceeding with the survey items.”
4. The authors should provide the reference or baseline level for the variables in Table 3, particularly for the categorical variables.
   a. Thank you for the feedback. We have edited the table so that it shows the reference group for categorical variables.

Best regards,

Tina Le, MPH

Herbert Wertheim School of Public Health

University of California, San Diego

1-714-804-8041
